# Use of Medicare’s New Reimbursement Codes for Cognitive Assessment and Care Planning, 2017-2018

**DOI:** 10.1001/jamanetworkopen.2021.25725

**Published:** 2021-09-17

**Authors:** Jing Li, Caroline Andy, Susan Mitchell

**Affiliations:** 1 Division of Health Policy and Economics, Department of Population Health Sciences, Weill Cornell Medical College, New York, New York; 2 Mailman School of Public Health, Columbia University, New York, New York; 3 Harvard Medical School, Boston, Massachusetts; 4 Marcus Institute for Aging Research, Hebrew Senior Life, Boston, Massachusetts; 5 Department of Medicine, Beth Israel Deaconess Medical Center, Boston, Massachusetts

## Abstract

This cross-sectional study assesses the use of Medicare's cognitive assessment and care planning codes in the first 2 years of their introduction.

## Introduction

Substantial barriers exist in timely detection of cognitive impairment, especially Alzheimer disease and related dementias (ADRD), impeding effective care planning and coordination among providers, patients and caregivers.^1,2^ To address these challenges, on January 1, 2017, Medicare began reimbursing clinicians for a comprehensive clinical visit for patients with cognitive impairment. Reimbursement requires cognition-focused evaluation, identification of caregivers and caregiver needs, and development, revision, or review of an Advance Care Plan.^3,4^ We provided the first assessment of the use of the new cognitive assessment and care planning (CACP) codes in the first 2 years of their introduction.

## Methods

The study was approved by the institutional review board of Weill Cornell Medical College. Informed consent was not obtained because only deidentified secondary data were used. We followed the Strengthening the Reporting of Observational Studies in Epidemiology (STROBE) reporting guideline.

We conducted a retrospective cross-sectional study using a 20% nationwide random sample of Medicare Part A and B claims and Medicare Beneficiary Summary File. Our study population included Medicare fee-for-service patients aged 65 years or older as of 2017 who were continuously enrolled in Medicare Part A and B between 2017 and 2018, or until death if they died during this period. Use of CACP was identified using *Current Procedural Terminology* code G0505 in 2017 and 99483 in 2018. We assessed the prevalence of patients with CACAP claims. Because patients with ADRD were the primary recipients of CACP, we stratified the claims by whether the patients had a diagnosis of ADRD in either 2017 or 2018 that had been identified by using the ADRD chronic condition indicator in the Medicare Beneficiary Summary File. Among patients with ADRD, we used multivariable logistic regression analysis to identify patient characteristics associated with having CACP in either 2017 or 2018. Patient characteristics included: age (ie, 65-74 years, 75-84 years, ≥85 years), gender, race, region of residence (ie, South, Midwest, West, Northeast), dual eligibility for Medicare and Medicaid, income quartile, and number of non-ADRD chronic conditions.^5^ *P* values were calculated using 2-sided *t* test, with .05 as the level of significance. Statistical analyses were performed from August 2020 to March 2021. SAS version 9.4 (SAS Institute) was used for all data analyses.

## Results

Our study population included 5 980 271 Medicare patients, and 895 726 of the patients (15.0%) had ADRD and 8355 of the patients (0.14%) had at least 1 CACP claim in either 2017 or 2018 (Table 1). Among patients with ADRD, 390 116 (43.5%) were aged 85 years or older, 358 500 (40.0%) resided in the South, 234 638 (26.2%) were Medicaid eligible, and 565 042 (63.1%) had at least 5 chronic conditions other than ADRD (Table 2).

**Table 1.** Use of CACP Among Medicare Fee-for-Service Patients in 2017 and 2018

Patients	Total patients, No.	Any CACP claims, No. (%)		Increase from 2017 to 2018, No. (%)^b^	CACP claims in 2017 or 2018, No. (%)^a^	CACP claims in both 2017 and 2018, No. (%)^c^
		In 2017^a^	In 2018^a^			
All	5 980 271	3893 (0.07)	5150 (0.09)	1257 (32.29)	8355 (0.14)	688 (8.23)
ADRD	895 726	2619 (0.29)	3639 (0.41)	1021 (38.95)	5796 (0.65)	462 (7.97)
Non-ADRD	5 084 545	1274 (0.03)	1511 (0.03)	237 (18.60)	2559 (0.05)	226 (8.83)

Abbreviations: ADRD, Alzheimer disease and related dementias; CACP, cognitive assessment and care planning.

^a^ The denominator is the number of total patients in column 2.

^b^ The denominator is the number of patients with CACP claims in 2017 as reported in column 3.

^c^ The denominator is the number of patients with CACP claims in either 2017 or 2018 as reported in column 6.

**Table 2.** Association Between Patient Characteristics and CACP Use Among Medicare Fee-for-Service Patients With ADRD, 2017-2018

Variables	No. (%)		Adjusted OR (95% CI)	*P* value
	Total (n = 895 726)	With CACP (n = 5796)		
Age				
65-74	163 812 (18.3)	1162 (20.0)	1 [Reference]	
75-84	341 798 (38.2)	2605 (44.9)	1.02 (0.95-1.09)	.65
≥85	390 116 (43.6)	2029 (35.0)	0.69 (0.64-0.74)	<.001
Gender				
Male	326 339 (36.4)	2213 (38.2)	1 [Reference]	
Female	569 387 (63.6)	3583 (61.8)	1.02 (0.97-1.08)	.40
Race				
Black	72 529 (8.1)	413 (7.1)	0.98 (0.88-1.08)	.68
White	775 571 (86.6)	5071 (87.5)	1 [Reference]	
Other^a^	47 626 (5.3)	312 (5.4)	1.07 (0.95-1.21)	.23
Region				
South	358 500 (40.0)	2564 (44.2)	1 [Reference]	
Midwest	205 614 (23.0)	625 (10.8)	0.42 (0.38-0.46)	<.001
West	191 721 (21.4)	1530 (26.4)	1.08 (1.00-1.15)	.03
Northeast	139 891 (15.6)	1077 (18.6)	1.01 (0.94-1.10)	.71
Dual eligibility for Medicare & Medicaid				
Nondual eligible	661 088 (73.8)	4770 (82.3)	1 [Reference]	
Dual eligible	234 638 (26.2)	1026 (17.7)	0.59 (0.55-0.63)	<.001
Quartile of median income of residence				
1 (lowest)	243 328 (27.2)	1299 (22.4)	1 [Reference]	
2	221 590 (24.7)	1249 (21.5)	1.07 (0.99-1.16)	.09
3	215 300 (24.0)	1485 (25.6)	1.26 (1.17-1.36)	<.001
4 (highest)	215 508 (24.1)	1763 (30.4)	1.41 (1.30-1.52)	<.001
Non-ADRD chronic conditions^b^				
0-1	71 066 (7.9)	405 (7.0)	1 [Reference]	
2-4	259 618 (29.0)	1622 (28.0)	1.12 (1.01-1.25)	.04
5-9	458 877 (51.2)	2980 (51.4)	1.23 (1.17-1.36)	<.001
≥10	106 165 (11.9)	789 (13.6)	1.45 (1.29-1.64)	<.001

Abbreviations: ADRD, Alzheimer disease and related dementias; CACP, cognitive assessment and care planning.

^a^ The category other includes individuals who identified as Asian, Hispanic, North American Native, or other races or ethnicities not specified in the data, and individuals of unknown race/ethnicity. We grouped these categories together because of small numbers in each group.

^b^ Number of non-ADRD chronic conditions was determined using the Centers for Medicare & Medicaid Services Chronic Condition Warehouse flags in the Medicare Beneficiary Summary File. Chronic conditions included acquired hypothyroidism, acute myocardial infarction, anemia, asthma, atrial fibrillation, benign prostatic hyperplasia, cataract, chronic kidney disease, chronic obstructive pulmonary disease and bronchiectasis, depression, diabetes, glaucoma, heart failure, hip/pelvic fracture, hyperlipidemia, hypertension, ischemic heart disease, osteoporosis, rheumatoid arthritis/osteoarthritis, stroke/transient ischemic attack, female/male breast cancer, colorectal cancer, prostate cancer, lung cancer, and endometrial cancer.

Among ADRD patients, 5796 patients (0.65%) had a CACP claims in either 2017 or 2018. The proportion patients with CACP claims increased from 2017 to 2018 for patients with ADRD and without ADRD (2017: patients with ADRD, 2619 [0.29%]; patients without ADRD, 1274 [0.03%]; 2018: patients with ADRD, 3639 [0.41%]; patients without ADRD, 1511 [0.03%]), and 688 (8.23%) who received any CACP services received them in both 2017 and 2018.

In an adjusted analysis, patients who were aged 85 years or older (odds ratio [OR] 0.69; 95% CI, 0.64-0.74), resided in the Midwest relative to the South (OR, 0.42; 95% CI, 0.38-0.46), and were eligible for Medicaid (OR, 0.59; 95% CI, 0.55-0.63) were associated with lower odds of having a CACP claim (Table 2). Patients were associated with higher odds of having CACP if they resided in the West relative to South, were in a higher income quartile, and had more chronic medical conditions.

## Discussion

There was a very low uptake of CACP claims in the first 2 years after its introduction, with disparities by patient age, number of chronic conditions, region, and income. Patients who are older may encounter more challenges in fulfilling the comprehensive evaluation required by CACP, and providers may prioritize CACP among those with more chronic conditions. Furthermore, providers in rural areas or small practices may be less likely to be aware of these codes, which may disproportionately affect patients in more remote geographic areas or patients with a lower socioeconomic status.^6^ However, it is possible that providers were billing components of the CACP service under separate reimbursement codes. Because of data limitations, we were unable to examine Medicare Advantage patients or include more recent years. However, our study suggests that increasing provider awareness of the new CACP service is important, perhaps through targeted continuing medical education at the facility level which may also address institution-specific challenges in billing CACP.
